# Systemic Inflammation Is Associated with Ovarian Follicular Dynamics during the Human Menstrual Cycle

**DOI:** 10.1371/journal.pone.0064807

**Published:** 2013-05-22

**Authors:** Kathryn B. H. Clancy, Angela R. Baerwald, Roger A. Pierson

**Affiliations:** 1 Laboratory for Evolutionary Endocrinology, Department of Anthropology, University of Illinois, Urbana-Champaign, Urbana, Illinois, United States of America; 2 Department of Obstetrics, Gynecology and Reproductive Sciences, University of Saskatchewan, Saskatoon, Saskatchewan, Canada; University of Wisconsin, United States of America

## Abstract

**Background:**

Ovarian processes and the timing of ovulation are important predictors of both female fertility and reproductive pathology. Multiple waves of antral follicular development have been documented during the menstrual cycle in women. However, the mechanisms underlying the development of follicular waves and their clinical significance are not fully understood. The objective of this study was to examine the relationship between C-reactive protein (CRP) and follicular waves in healthy women. We wanted to determine whether follicular wave dynamics influence systemic inflammation, as ovarian activity increases local inflammatory processes and blood flow. We tested the hypothesis that women with 3 follicular waves would have higher CRP concentrations than those with 2 waves. We further hypothesized that a greater number of major waves (those with a dominant follicle) would be positively associated with CRP.

**Methods/Principal Findings:**

Thirty-nine healthy women underwent daily transvaginal ultrasound examinations for one interovulatory interval, as part of an earlier study. Serum was collected every 3 days during the interovulatory interval (IOI). Enzyme-linked immunosorbent assays were conducted to quantify serum CRP concentrations. Women with 3 waves had higher average log CRP concentrations (n = 14, −0.43±0.35) over the IOI than those with 2 waves (n = 25, −0.82±0.47, p = 02). Average log CRP concentrations were greater in women with 3 (0.30±0.31) versus 1 (−0.71±0.55) or 2 (−0.91±0.47) major waves (p = 0.03). Greater average CRP over the IOI was attributed to greater CRP in the follicular, but not the luteal phase, of the IOI.

**Conclusions/Significance:**

A greater number of total antral follicular waves, in particular major waves, corresponded to greater serum concentrations of CRP. These findings suggest that women with a greater number of follicular waves exhibit greater tissue remodeling and therefore greater local and systemic inflammation.

## Introduction

Multiple waves of antral follicular development have been documented histologically, ultrasonographically, and endocrinologically during the menstrual cycle [1]–[5]. Follicular waves have been shown to reflect normal ovarian function in healthy women of reproductive age [2], [6] and advanced reproductive age [7]–[9]. Follicular waves have also been detected in women undergoing ovarian stimulation for the treatment of infertility [10] and during the hormone free interval in women using hormonal contraception [11]. Observations of antral follicular waves in women are similar to those previously documented in animal species, including cattle [12]–[15], mares [16]–[18], sheep and goats [19], [20], llamas and alpacas [21], [22], musk oxen [23], water buffalo [24], deer and wapiti [25], [26], as well as non-human primates [27], [28]. Knowledge about the dynamics of antral folliculogenesis in women is fundamental for understanding the normal variation in human ovarian physiology and fertility [29]–[32].

Antral follicle dynamics vary between women. In one study, 34/50 healthy women with a history of regular cycles were shown to develop 2 waves (68%) and 16/50 developed 3 waves (32%) during an interovulatory interval (IOI) [2], [6]. The final wave of the IOI was ovulatory, while all preceding waves were anovulatory. In most women, the anovulatory waves were ‘minor’, in which a dominant follicle did not develop [6]. However, in 22–48% of women, one or two anovulatory major waves (i.e., in which a dominant follicle developed) were shown to precede the ovulatory wave [6]–[9]. Women with 3 waves had later pre-ovulatory rises in estradiol, LH, FSH and longer cycles compared to women with 2 waves [6]. Each wave emerged in association with a rise in both FSH and inhibin B [6], [8], [9]. Research conducted to date has been an important step in characterizing antral follicular wave dynamics in women. However, continued research is needed to further characterize the endocrine regulation of follicular wave dynamics in women and to more fully understand the clinical significance of multiple follicular waves during the menstrual cycle.

Relationships between the gonadotropins, ovarian steroid hormones and inflammatory markers have been established in humans and animal models [33]–[36]. The development of dominant follicles throughout the menstrual cycle involves increases in ovarian blood flow and tissue remodeling. Thus, it is plausible that systemic inflammation may be a correlate of follicular wave dynamics. C-reactive protein (CRP) is a 224-residue protein belonging to the pentraxin family that often serves as a biomarker for systemic inflammation [37]. CRP is found in the blood, and rises in response to inflammatory stimuli to activate the complement system. It is largely produced by the liver in response to increases in production of IL-6 from macrophages and adipocytes. CRP indicates systemic inflammation and modulates in response to illness, parasite load, as well as changes in diet and weight [38]–[41]. CRP tends to be higher in women than men [42]–[44], and this gender difference in CRP production appears to be consistent across European American, African American and Latino American groups [44]. CRP is also more strongly associated with adiposity in women compared to men [45].

Variations in inflammatory markers have been described in reference to the menstrual cycle. Serum CRP concentrations have been shown to be greatest in women at midcycle in one study [46]. These findings were attributed to inflammatory processes occurring within the ovary at the time of ovulation [35], [47], [48]. CRP concentrations have also been shown to be greater in women during the mid-luteal phase (period of high progesterone concentrations), compared to the mid-follicular phase (a period of relatively lower ovarian steroid hormone concentrations) of the menstrual cycle [46]. CRP has also been shown to be negatively associated with estradiol [49]–[51] and progesterone [51], and also positively with progesterone [50]. Increased CRP has also been observed during menses and attributed to inflammatory processes in the uterus associated with menstruation [46], [49], [50]. In contrast, Wunder et al [52] found no relationship between CRP and ovarian hormones during the menstrual cycle. Significant methodological differences characterize these studies, making it a challenge to discern the meaning behind the variable findings.

CRP has been associated with hormone production and/or clinical outcomes in women administered exogenous gonadotropins and/or steroid hormones. Administration of exogenous estradiol in women using hormonal contraceptives was positively associated with CRP [53]–[59]. Similarly, supraphysiologic endogenous estradiol concentrations in women undergoing assisted reproductive technologies (ART) were positively correlated with CRP [60], [61]. Women undergoing IVF were more likely to conceive when a rise in serum CRP was detected within 1–2 weeks of oocyte retrieval [60], [61]. CRP was also elevated at 4 weeks gestation in a sample of women who conceived successfully with IVF [62]. In addition, intentional local endometrial inflammation during blastocyst transfer following IVF has been shown to improve pregnancy outcomes [63]. Increased maternal CRP may be associated with protection from pregnancy loss, particularly in losses greater than 12 weeks gestation [64]. Thus, an inflammatory response has been suggested to be a positive signal for implantation; however, too much (or too little) inflammation may indicate pathology [62], [65].

Collectively, these data suggest a connection between CRP and female reproductive function. The precise relationships and underlying mechanisms of action, with particular reference to ovarian function, however, are not understood. Research conducted thus far has characterized CRP concentrations in relation to menstrual cycle phase and hormone production, with variable sampling methodologies. However, no studies have been conducted to date to elucidate the potential relationships between CRP and ovarian follicular dynamics, the antecedent to hormone production. It is plausible that a greater number of follicle waves during the menstrual cycle may be associated with a greater degree of inflammation. Because the process of ovulation involves significant tissue remodeling, we anticipate that follicular wave activity produces a local inflammatory response that leads to an increase in systemic inflammation. A greater understanding about potential associations between CRP concentrations and ovarian follicular dynamics may provide insight into the physiologic origins of inflammation and/or ovarian dysfunction. Furthermore, studies of this nature may provide knowledge about the factors influencing normal variations in CRP concentrations during the menstrual cycle within and among women.

The objective of the present study was to determine whether serum CRP concentrations were associated with follicular wave dynamics during the human menstrual cycle. The following hypotheses were tested: 1) individuals with 3 follicular waves during an IOI would have higher CRP concentrations than those with 2 waves, and 2) individuals with a greater number of major waves would have greater CRP concentrations compared to women with few major waves.

## Materials and Methods

### Study Population

Serum samples, collected as part of an earlier study in the Department of Obstetrics, Gynecology and Reproductive Sciences at the University of Saskatchewan, Canada [2] were analyzed in the present study. The study population consisted of 39 healthy women with a history of regular menstrual cycles (i.e., menses every 25–35 days) and normal reproductive functioning. In the original study, participants were recruited using advertisements placed at the University of Saskatchewan and Saskatoon Health Region. Only 39/50 datasets obtained from the original study were available for evaluation in this secondary set of analyses due to normal sample loss from completed assays in previous work. Participants were assessed, by history and physical examination, to be healthy women of reproductive age (mean ± SD  = 28.0±6.9 yr, range  = 19–43 yr). Women who were currently pregnant, pregnant or lactating within the past 12 months, had used hormonal contraception within 3 months of enrollment, had a history of irregular menstrual cycles, or were taking medication(s) known or suspected to interfere with reproductive function were not eligible to participate. Informed consent was obtained from all women prior to initiating study procedures. The study protocol was approved by the Institutional Review Board of the University of Saskatchewan and all participants gave written informed consent. The University of Illinois Institutional Review Board approved the secondary research (additional laboratory and statistical analyses).

### Study Methods

The numbers and diameters of all follicles ≥2 mm were recorded daily using transvaginal ultrasonography for one complete IOI, as previously described [2]. An IOI was defined as the period from one ovulation to the subsequent ovulation. Ovulation was identified ultrasonographically as the absence of an ovarian follicle that had been observed on the previous day and the subsequent formation of a corpus luteum. Ovulation was also confirmed endocrinologically as a rise in serum progesterone concentration. Follicular waves were characterized by an increase and subsequent decrease in the number of follicles ≥5 mm, occurring in association with the growth of at least 2 follicles to ≥6 mm. Major (+) waves were characterized as those in which a dominant follicle was selected for preferential growth. Dominant follicles were characterized as follicles which developed to at least 10 mm in diameter, and their continued growth exceeded the next largest follicle by ≥2 mm. Minor (−) waves were characterized as those in which a dominant follicle was not detected. Five different wave patterns of follicle growth were documented: − +, + +, − − +, − + +, + + +. The number and pattern of follicle wave development across the IOI were tabulated. The luteal phase was defined as the time period from the day of ovulation #1 to the day before menses. The follicular phase was defined as the time period from the first day of menses to the day before ovulation #2. Wave emergence was defined as the day that the dominant follicle was retrospectively identified at a diameter of 4–5 mm.

Blood samples were collected every 3 days during the IOI in a stratified manner among women so that each day of the IOI was represented, as previously described [2]. Samples were shipped on dry ice to the Laboratory for Evolutionary Endocrinology at the University of Illinois, Urbana-Champaign, where they were stored at −20°C. Assays for CRP were conducted using high-sensitivity enzyme immunoassay kits (Helica Biosystems Inc, Fullerton CA USA: cat no. 961CRP01H-96). Interassay co-efficients of variation were 17.5% for the highest CRP standard and 12.6% for the lowest standard. The detection limit for the CRP assay was 0.20 ng/mL.

### Statistical Analyses

CRP concentrations throughout the IOI were not normally distributed, and thus were log-transformed.

Parametric tests were used to evaluate log-transformed CRP concentrations during the menstrual cycle (GraphPad Prism 5; La Jolla, CA USA). Unpaired t-tests were used to compare mean CRP concentrations at various time points during the IOI among women with 2 or 3 follicular waves. One-way analyses of variance (ANOVA) were used to compare participant demographics and CRP among women with different wave patterns.

Two-way repeated measures Analyses of Variance (ANOVA) were used to analyze serial changes in CRP concentrations (PROC MIXED; SAS Version 9, Cary, NC). Data were aligned at first ovulation and at first day of menses. Alpha was set at 0.05. Sample sizes of 14 per group were estimated to provided 80% power to detect differences in log CRP concentrations of 0.44 between groups. Significance was set at 0.05. Statistics were conducted on log-transformed data; however, figures were created using untransformed CRP concentrations to represent physiologically meaningful values.

## Results

### Participant Demographics

Participant demographics were compared in women with different numbers and patterns of follicle wave development in Table 1. Subjects were 19 to 42 years old (mean age ± SD, 28.10±7.51 years) with a mean BMI of 26.35±4.11, mean gravidity of 1±2 pregnancies (range  = 0–6), and mean parity of 1±1 births (range  = 0–5). No differences in age, gravidity, or parity were detected in women with 2 versus 3 follicular waves (p>0.05). Women with 2 waves (25.19±4.00) had a lower BMI than those with 3 waves (28.42±3.54, p = 0.02).

**Table 1:** Differences in age, gravidity, parity and BMI between women with 2 versus 3 waves, among the 5 follicular wave patterns, and among those with different numbers of waves emerging in the follicular phase.

Group	n	Age	Gravidity	Parity	BMI
2 waves	25	28.52±8.33	1.16±1.91	1.04±1.67	25.19±4.00
3 waves	14	28.05±5.98	1.07±1.82	0.86±1.35	28.42±3.54
p-value		0.85	0.88	0.73	**0.02**
−+ pattern	21	27.92±7.23	0.59±1.12^a^	0.55±1.02^a^	25.29±4.57
++ pattern	4	30.86±9.41	3.40±2.70^b^	2.80±2.59^b^	26.68±2.98
+++ pattern	3	25.36±6.27	1.00±1.73^a,b^	1.00±1.73^a,b^	27.71±1.18
−++ pattern	2	30.26±10.49	1.50±2.12^a,b^	1.00±1.41^a,b^	27.32±4.47
−−+ pattern	9	28.45±5.52	1.00±2.00^a,b^	0.78±1.39^a,b^	28.90±4.10
p-value		0.63	**0.01**	**0.01**	0.14

Values are shown as the mean ± standard deviation. Within columns, values with different superscripts are different (p<0.05).

There were no differences in age or BMI among women with different major and minor patterns of follicular waves (p>0.05). Higher gravidity and parity were reported in the ++ (gravidity: 3.40±2.70; parity: 2.80±2.59) group compared to the −+ (gravidity: 0.59±1.12; parity: 0.55±1.02) and −++ (gravidity: 1.50±2.12; parity: 1.00±1.41) groups (p = 0.01). Neither BMI (p = 0.68, r^2^ = 0.005), age (p = 0.72, r^2^ = 0.004), nor parity (p = 0.56, r^2^ = 0.009) correlated with log-transformed average CRP concentrations.

Menstrual cycle characteristics were compared between women with different numbers and patterns of follicle waves. Women with 3 waves had a longer follicular phase and longer IOI compared to women with 2 waves. There were also similar statistically significant differences in follicular phase (p = 0.01) and IOI (p = 0.02) among the five wave patterns, where post-tests revealed that the −++ group had a significantly longer follicular phase than the −+ group. (Table 2).

**Table 2:** Differences in IOI, follicular and luteal phase lengths among the different wave patterns.

Group	n	Follicular phase length	Luteal phase length	Interovulatory interval
2 waves	25	14.07±1.67	13.28±1.39	27.34±2.30
3 waves	14	16.25±2.93	13.75±1.06	30.00±2.17
p-value		**0.004**	0.30	**0.002**
−+ pattern	21	13.96±1.72^a^	13.36±1.41	27.32±2.34
++ pattern	4	14.75±1.26^a,b^	12.75±1.26	27.50±2.38
+++ pattern	3	16.50±2.12^a,b^	13.50±0.71	30.00±1.41
−++ pattern	2	19.00±4.24^b^	13.00±1.41	32.00±2.83
−−+ pattern	9	15.50±2.73^a,b^	14.00±1.07	29.50±2.14
p-value		**0.01**	0.59	**0.02**

Values are shown as the mean ± standard deviation. Within columns, values with different superscripts are different (p<0.05).

### CRP and ovarian hormones

All CRP concentrations were log-transformed and thus unitless. Log-transformed CRP averaged over the IOI was not correlated with follicular phase Luteinizing Hormone (LH) (p = 0.18, r^2^ = 0.05), Follicle Stimulating Hormone (FSH) (p = 0.68, r^2^ = 0.005), estradiol (p = 0.64, r^2^ = 0.006), or luteal phase progesterone (p = 0.36, r^2^ = 0.02).

CRP concentrations were not different when averaged over the follicular (−0.68±0.58) versus luteal phases of the IOI (−0.69±0.52; p = 0.73, r^2^ = 0.003).

### CRP and follicular waves

**Figure 1:**
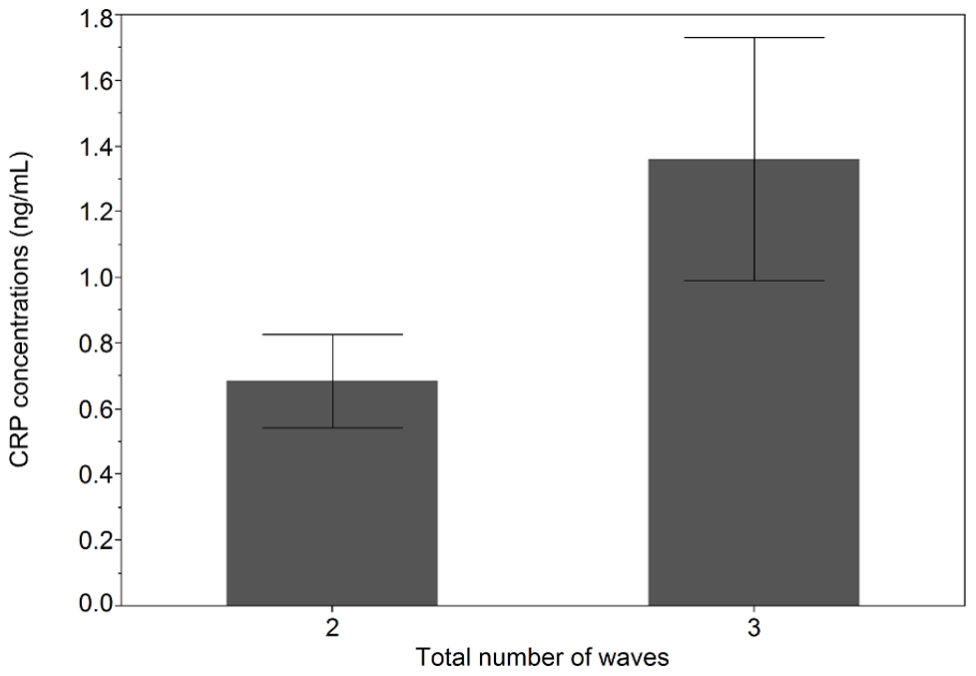
Mean CRP concentrations over the IOI in women with two or three follicle waves. Non-transformed data are shown as the mean ± SEM.

Women with 3 follicle waves had a higher mean CRP concentration over the IOI (n = 14, −0.43±0.35) compared to women with 2 follicle waves (n = 25, −0.82±0.47, p = 0.02) (Figure 1). Mean CRP concentrations over the follicular phase were higher in women with 3 (−0.56±0.66) versus 2 (−0.76±0.48) follicle waves (p = 0.02, Table 3). No differences in mean luteal phase CRP concentrations were detected between groups (p = 0.11, Table 3).

**Table 3:** CRP concentrations within each phase of the IOI and within each follicle wave compared among women with different numbers and patterns of follicular waves.

Group	n	Luteal Phase	Follicular Phase
2 waves	25	−0.79±0.60	−0.76±0.48
3 waves	14	−0.52±0.40	−0.56±0.66
p-value		0.11	**0.02**
−+ pattern	21	−0.74±0.60	−0.75±0.49^a^
++ pattern	4	−1.10±0.50	−0.86±0.48^a^
+++ pattern	3	−0.75±0.58	0.23±0.23^b^
−++ pattern	2	−0.47±0.05	−0.69±0.36^a,b^
−−+ pattern	9	−0.46±0.43	−0.74±0.66^a,b^
p-value		0.33	**0.05**

Values are shown as the mean ± standard deviation. Within columns, values with different superscripts show a trend towards difference (p<0.10).

**Figure 2:**
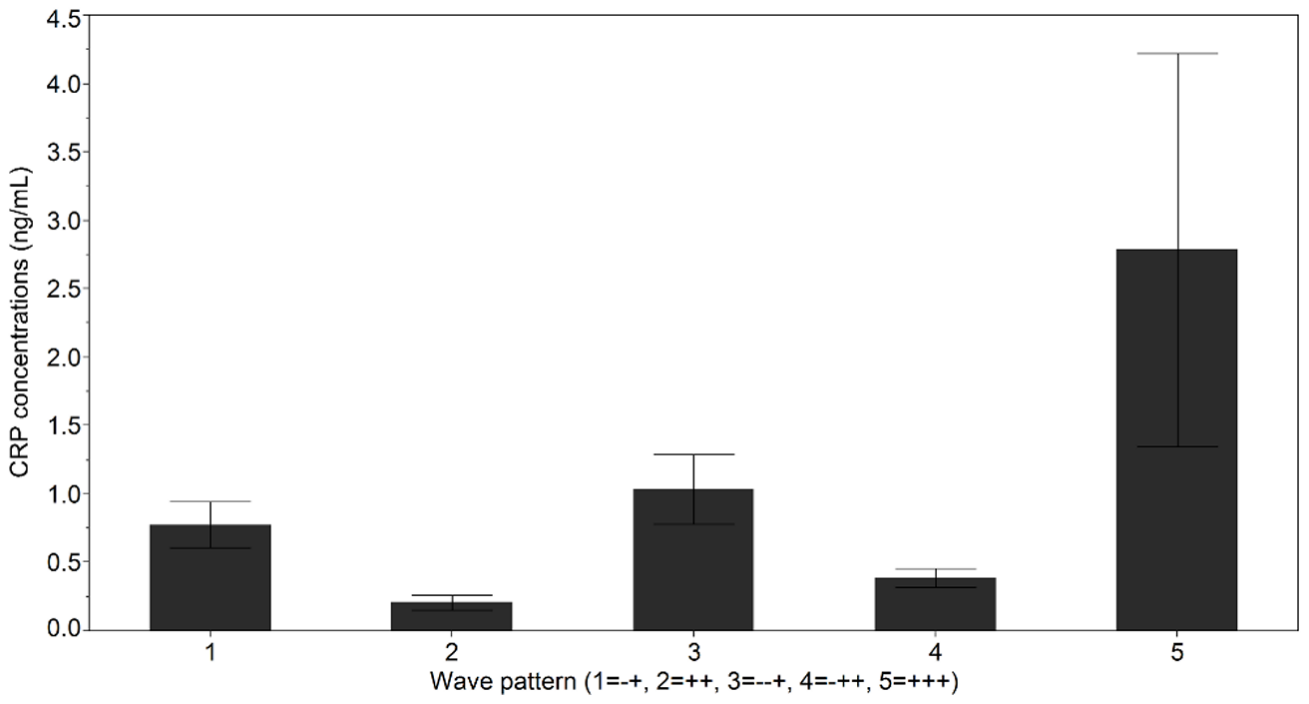
Mean CRP concentrations over the IOI in women with different patterns of major and minor follicle development. Two waves: −+ and ++; three waves: −−+, −++ and +++. Non-transformed data are shown as the mean ± SEM.

**Figure 3:**
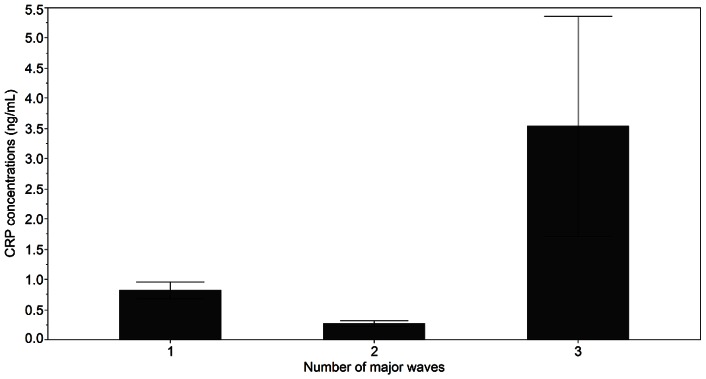
Mean CRP concentrations over the IOI in women with one, two or three major waves, irrespective of total number of waves. Non-transformed data are shown as the mean ± SEM.

Mean CRP concentrations over the IOI were also different among the five wave patterns, where CRP was greater in women with +++ compared to all other groups (p = 0.05, Figure 2). Follicular phase CRP was also different among the five wave patterns (p = 0.05), with a tendency for higher CRP in the +++ versus −+ and ++ group (0.05<p<0.10). Mean CRP concentrations over the IOI were greater in women with 3 (0.30±0.31) versus 1 (−0.71±0.55) or 2 (−0.91±0.47) major waves (p = 0.03, Figure 3).

**Figure 4:**
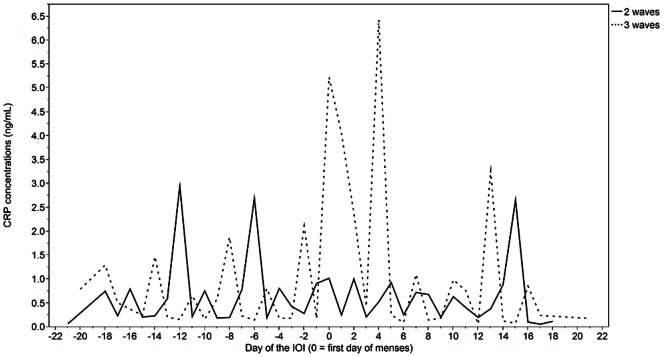
CRP concentrations across the IOI in women with two versus three follicle waves. Non-transformed data are shown.

Serial changes in CRP over the cycle are shown in Figure 4. CRP appeared to fluctuate every 2–6 days during the IOI. Pre-ovulatory rises in CRP were detected in women with either 2 or 3 follicle waves during the IOI. Lower amplitude fluctuations in luteal phase CRP were detected in women with 3 versus 2 follicular waves (day effect: p = 0.49, wave effect: p = 0.01, day*wave effect: p = 0.33). On the contrary, higher amplitude fluctuations in early follicular phase CRP were detected in women with 3 versus 2 waves (day effect: p = 0.11, wave effect: p<0.0001, day*wave effect: p = 0.34). Changes in CRP concentrations over the luteal (adjusted r^2^ = 0.03), and follicular (adjusted r^2^ = 0.46), phase were positively correlated in women with 3 versus 2 waves.

**Figure 5:**
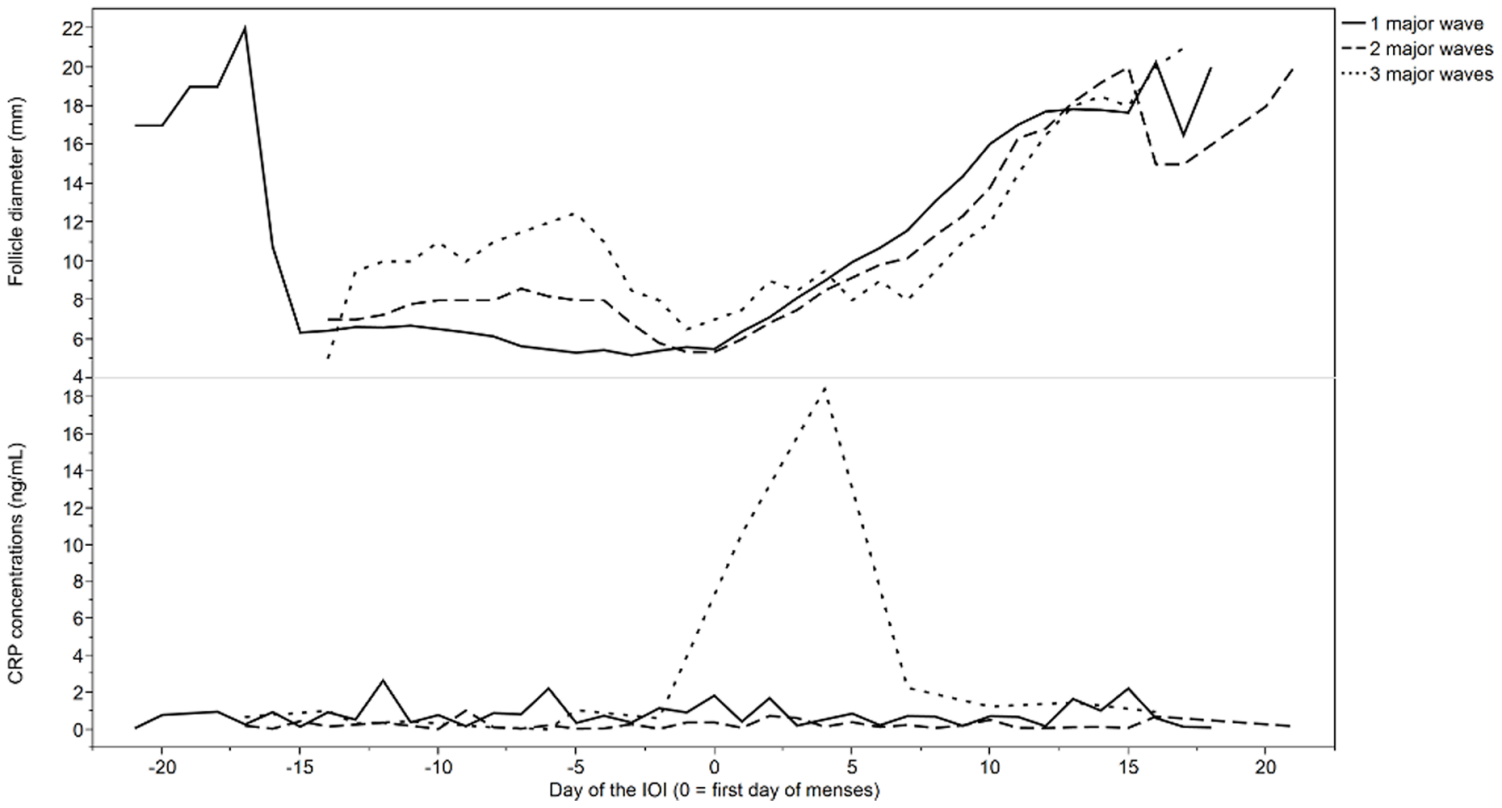
Follicle diameter and CRP concentrations are aligned by menses through the IOI to determine potential time-dependence of relationships between follicular waves and inflammation. A) Mean follicle diameter in women with 1, 2 and 3 major waves. B) CRP concentrations in women with 1, 2 and 3 major waves. Non-transformed data are shown.

Serial changes in CRP concentrations over the IOI in women with 1, 2 or 3 major waves in association with changes in dominant follicle growth are shown in Figure 5. A prominent rise and fall in follicular phase CRP was detected in women with 3 but not 2 or 1 major waves (Figure 5B; day effect: p = 0.10, major wave effect: p = 0.19, day*major wave effect: p = 0.002). The prominent rise and fall in CRP occurred in association with the development of the 2^nd^ of the 3 major waves of the IOI (Figure 5A).

A day*major wave effect and trend toward a day effect were also found when comparing women among the five wave patterns, again largely a result of the early follicular spike in CRP in the +++ group compared to the −+ and ++ groups (day effect p = 0.10, pattern*day effect p = 0.04, pattern effect p = 0.28).

## Discussion

In this study, we examined the relationship between ovarian follicular wave dynamics and inflammatory processes represented by circulating CRP concentrations. Women with 3 follicular waves were found to have greater serum CRP concentrations during the menstrual cycle, in particular during the follicular phase, compared to women with 2 waves. Furthermore, women with 3 major waves during the IOI had greater CRP concentrations than those with 1 or 2 major waves. Collectively, these findings suggest that women who develop multiple major waves of follicle development during a given menstrual cycle exhibit a greater degree of systemic inflammation. Thus, our hypotheses were supported.

The relationships between local (e.g., ovarian) and systemic inflammatory processes, and how they influence each other in relation to reproductive function are not fully understood. Numerous environmental factors have been shown to correlate with CRP, including diet composition [66], [67], psychosocial stressors [68], [69], adiposity [39], [45], [70]–[72] and pathogen exposure during infancy [38], [39]. These environmental factors could be an external factor influencing systemic inflammation, which in turn influences reproductive function.

However, local ovarian processes can also drive inflammation. In a sample of women during a controlled ovarian hyperstimulation protocol for an IVF embryo transfer, serum CRP was higher on hCG administration and oocyte pick-up days compared to the suppression stage [59], [61]. Our findings support the notion that dominant follicle development leads to follicular vascularity which causes local ovarian inflammation, contributing to systemic inflammation.

Previous researchers have reported a rise in CRP at the time of menses [46], [50], more specifically a 50% increase in the variance in CRP concentrations at menses compared to other phases of the cycle [50]. This rise was attributed to endometrial cell turnover at the time of menses. However, in our study, we were able to show that a rise in CRP during the late luteal/early follicular phase was associated with follicle development, rather than menstruation in 30% of women evaluated (i.e., those with 3 follicle waves). This finding was even more obvious in the 8% of women evaluated that developed 3 major follicle waves. Thus, our data are novel in that they suggest an ovarian, versus uterine, origin of systemic inflammation during the menstrual cycle.

A greater understanding about the relationships between local inflammation, systemic inflammation and ovarian follicle wave dynamics may contribute to our understanding of variation in ovarian function among women. Ultrasound image attributes of dominant follicles in ovulatory versus anovulatory waves have been shown to differ [73]. More specifically, mean numerical pixel value and pixel heterogeneity were greater in dominant anovulatory follicles compared to dominant ovulatory follicles. It is plausible that dominant follicles from anovulatory versus ovulatory waves in women may exhibit differences in functional status, similar to those documented in animal models. Continued research is required to characterize the functional status of follicles developing in 2 versus 3 waves during the cycle as well as in ovulatory versus anovulatory waves and their reproductive implications.

There were a few limitations to our study. First, CRP is not a very distinct signal. It is a correlate of many kinds of external inflammatory stressors and internal processes; therefore, it is challenging to confirm the directionality of any relationships between physiological or environmental factors and CRP. Future work is required to elucidate the relationship between ovarian function and inflammatory processes, with the inclusion of additional means for assessing various sources of inflammation. The addition of local IL-6 measurements and ovarian vascularity, as well as sensitive anthropometry that considers body fat and participants' psychosocial stress, could be useful in parsing the origins of inflammatory variation. Second, this study was not designed to assess the potential effects of age or BMI on ovarian function and CRP concentrations. CRP concentrations did not correlate with age or BMI in the present study as in other reports [39], [43], [71], [74]. However, BMI and CRP were higher in women with 3 versus 2 waves. Thus, it is possible that interplay between systemic inflammation, energy status, and ovarian function exists. Further research is ongoing in our laboratory to elucidate the potential effects of energy status on ovarian follicular wave dynamics, particularly in women with Polycystic Ovarian Syndrome. Third, the method of serum sample collection every three days throughout the IOI, though stratified, may not capture all the variation in CRP. Fluctuations in CRP were reported throughout the menstrual cycle in all women evaluated; however, the clinical significance of these fluctuations is not fully understood. Future studies that include more frequent sampling may better characterize acute changes in CRP and correlations with follicle dynamics and changes in hormone production during the menstrual cycle.

The study of inflammatory processes in the ovary is a new area of research. We found that CRP was higher in women with a greater number of follicular waves, in particular a greater number of major waves. The development of a greater number of major waves during the menstrual cycle has been associated with a longer cycle length [6] and a greater number of antral follicles [7]–[9]. Thus, it is possible that the development of more major waves (and therefore multiple dominant follicles) may indicate a greater ovarian reserve and thus greater capacity for developing follicles capable of reaching ovulatory status. The greater local (and resultant systemic) inflammation may be the natural consequence of more dominant and vascularized follicles developing throughout the cycle. Luteal influences on follicular wave dynamics are not yet understood. Anovulatory major waves preceding the ovulatory wave could result from a breakdown in regulatory (e.g. luteal) processes that may otherwise prevent dominant follicles from developing in the luteal and/or early follicular phase of the cycle. High CRP among women with greater follicle wave activity may indicate that a pathologic state of multiple dominant follicle development results in greater local and systemic inflammation. Further investigations are required to test these hypotheses. Continued research is also required to discern environmental factors that may influence ovarian follicular development and associated inflammation. Should variation in wave activity be found to cause variation in systemic inflammation, follicle wave patterns will need to be controlled for in future studies of inflammatory processes among normo-ovulatory women.
